# Impact of a Weight-Loss Rehabilitation Program on Sleep Apnea Risk and Subjective Sleepiness in Patients with Overweight/Obesity: The DietSleep Study

**DOI:** 10.3390/jcm11236890

**Published:** 2022-11-22

**Authors:** Sébastien Bailly, Odile Fabre, Mallory Cals-Maurette, Laurent Pantagis, Robin Terrail, Rémy Legrand, Arne Astrup, Jean-Louis Pépin

**Affiliations:** 1 Grenoble Alpes University, INSERM U1300, Grenoble Alpes University Hospital, 38000 Grenoble, France; 2 Groupe Éthique et Santé, Actiburo 1, Bâtiment A—100 Chemin de l’Aumône Vieille, 13400 Aubagne, France; 3 Department of Obesity and Nutritional Sciences, Novo Nordisk Foundation, 2900 Hellerup, Denmark

**Keywords:** weight loss, obstructive sleep apnea, sleepiness

## Abstract

Obstructive sleep apnea (OSA) is one of the most frequent chronic diseases, and comorbid obesity occurs in more than 60% of cases. Variations in body weight influence both OSA severity and OSA-related symptoms. We prospectively assessed the impact of a weight-loss program using the Berlin score to reflect OSA risk, and we also used the Epworth Sleepiness Scale (ESS) to assess daytime sleepiness. DietSleep was a prospective multicentric cohort study investigating OSA risk and daytime sleepiness before and after weight-loss intervention. One hundred and twenty-seven patients were included (initial OSA risk 36%), most of whom were women (85.8%) with a median body mass index (BMI) of 29.7 kg/m^2^, and the interquartile range was (27.6; 34). The diet-based weight-loss program induced a median decrease in BMI of 3.7 kg/m^2^ (−5; −2.9) (body weight~12.1% (−16.0; −8.8)) over a period of 171 days (114; 269). Changes in anthropometric values were similar regarding OSA risk after adjusting for initial values. Berlin scores significantly improved from 3 (1; 5) to 1 (0; 2), *p* < 0.01; the proportion of patients with a Berlin score ≥2 decreased from 36% to 7% after the intervention. The proportion of patients with ESS ≥11 decreased from 13% to 2%. These results confirm that a weight-loss program produces clinically relevant weight loss and a significant improvement in both OSA and subjective daytime sleepiness.

## 1. Introduction

While it is often underdiagnosed, obstructive sleep apnea (OSA) represents a highly prevalent chronic disease that [1] contributes to the progression and aggregation of cardiovascular and metabolic comorbidities [2,3,4]. A bidirectional relationship exists between body weight, obesity, and OSA [2,5]. The prevalence of OSA is up to 40% in obese adults, and the risk increases with body mass index (BMI) [5]. OSA also remains largely undiagnosed in this particular population [6,7].

Disturbed sleep habits, such as both shorter and longer sleep times, are known to be associated with overweight and obesity [8,9]. In a prospective cohort of moderately overweight men and women, increasing weight over time aggravated OSA severity [10]. More recently, it has been shown that weight loss is associated with specific changes at the pharyngeal level, with a reduction in fat proportion in upper-airway tissues contributing to a reduction in OSA severity [11].

Although the prevalence of overweight and obesity has increased worldwide [12], and is considered to be an epidemic, there are actionable risk factors that can be targeted to improve the severity of OSA. Continuous positive airway pressure (CPAP) is the first-line therapy for OSA, but treatment adherence remains an important issue. The rate of CPAP termination observed in a nationwide study was 47% in the 3 years following the initiation of CPAP [13], and it was associated with a higher rate of cardiovascular events and mortality [14]. The effect of CPAP on cardiometabolic risk reduction shows a higher potential when associated with weight-loss-reduction programs [15,16,17].

Weight loss achieved by lifestyle interventions or bariatric surgery is associated with a decrease in OSA severity, and it improves both OSA-related symptoms and OSA patient-reported outcomes [18]. Alternatives to CPAP are therefore highly desirable, and non-CPAP therapies, including weight-loss management in overweight and obese OSA patients, are recommended by European and U.S. guidelines [18,19]. Accordingly, the American Thoracic Society recommends that sleep physicians regularly assess weight and include overweight and obese OSA patients in comprehensive lifestyle interventions that incorporate weight loss and physical activity [18].

However, an assessment of OSA severity is not routinely included in the baseline evaluation of patients with overweight or obesity who initiate weight-loss programs. Risk score questionnaires have been developed to assess the probability of suffering from OSA [20]. One of the most widely used tools is the Berlin questionnaire [21], which aims to identify patients at a high risk of OSA based on the burden of symptoms, including snoring severity and comorbidities [22,23].

The diet-based RNPC weight-loss rehabilitation program (for Rééducation Nutritionnelle et Psycho-Comportementale, i.e., Nutritional and Psycho-Behavioral Rehabilitation) has demonstrated an ability to achieve significant weight loss in large real-life cohorts [24,25]; however, it has never been implemented in relation to improvements in OSA risk or the impact of OSA-related symptoms.

The objective of this study was to prospectively investigate the impact of a weight-loss program on OSA risk and subjective daytime sleepiness, as measured by the Berlin score and the ESS, respectively, in patients without known OSA. The Berlin score was chosen due to its performance in the general population and because it is easy for dieticians to use as a screening tool compared to an objective sleep study.

## 2. Materials and Methods

### 2.1. Study Design

**Figure 1:**
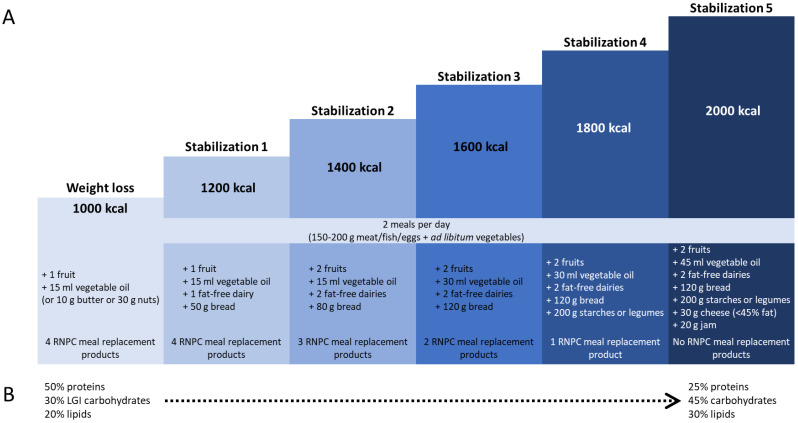
The RNPC program, from weight loss to balanced weight. (**A**): Example of a diet plan for a man with daily energy requirements estimated at 2000 kcal by Black formula. Energy intake and food consumed daily increase throughout the RNPC program, while the number of RNPC meal replacement products decreases. (**B**): Evolution of macronutrient distribution from weight loss to maintenance phase.

The DietSleep study was a prospective multicenter cohort study conducted in six RNPC centers in France that are involved in a standardized national weight-loss program: the RNPC program. This program has been described extensively elsewhere [24,25,26]. Briefly, the RNPC program is a three-stage weight-loss program routinely performed in 79 centers across France. The RNPC diet is composed of two meals per day, including vegetables (ad libitum), one fruit (among a list of low-calorie and low-glycemic-index vegetables and fruits provided to the patients by the dietician), animal proteins (from meat, fish, eggs, or shellfish; 100–200 g/meal), and commercially available meal supplements (these do not replace a meal), as quantified through four units for men and three for women. Additionally, snacks are provided (biscuits, cereal bars, bread, crackers, soups, omelets, drinks, and desserts) that are ready to eat or easy to prepare, and the calorie content should not exceed 200 kcal/unit. The patients can eat whenever they want during the day (Figure 1).

Participants are referred to the RNPC program by their clinician, essentially general practitioners, to achieve the primary objective of weight loss. The first two stages of the program are: (1) a weight-loss phase, during which rapid weight loss is achieved (two to six months), and (2) a stabilization phase, during which energy intake is gradually increased with a duration depending on the weight-loss phase (i.e., 1 week per kg lost during the initial phase). The program is followed-up with a maintenance phase during which energy balance is achieved.

In the maintenance period, participants can return to the program whenever necessary. Face-to-face consultations with RNPC dieticians are scheduled once every two weeks during the first two stages (weight loss and stabilization), and they then become optional during the maintenance phase. Ethical approval for the study was issued by the CPP Sud Méditerranée III.

### 2.2. Study Objectives

The co-primary objectives of the study were to compare the evolution of OSA risk and subjective daytime sleepiness between the initiation and the end of the weight-loss phase using the RNPC weight-loss program.

The secondary objectives were to assess the relationship between the amplitude of weight-loss reduction and anthropometric measures of obesity, office blood pressure, and medication prescription evolution between the initiation and the end of the weight-loss phase using the RNPC program in specific subgroups either at risk or not at risk of OSA.

### 2.3. Study Population

Patient inclusions started in March 2019 and adhered to the following criteria: initial age≥ 18 years, BMI ≥ 25 kg/m^2^, and starting an RNPC program. Due to the COVID-19 pandemic, a significant delay occurred until end of inclusions in March 2021, and withdrawal from the study happened during the pandemic period (*n* = 115). Only age and sex were available to compare the included patients and patients lost to follow-up. Finally, for this study, only patients without diagnosed OSA at the start of the RNPC program were included.

### 2.4. Data Collection and Measures

The following data were systematically collected via an electronic medical record in each participating center: patient characteristics (age, gender), tobacco consumption, and anthropometric measures (weight, BMI, waist circumferences, percentages of fat mass, muscle mass, and body water). Anthropometric measures were collected during each in-patient follow-up visit with a calibrated bioelectrical impedance scale (BG42, Beurer, Ulm, Germany). Waist circumference was measured to the midpoint between the lower border of the rib cage and the iliac crest. For participants with abdominal adiposity with no visible natural waist, the measurement was taken approximately 2–5 cm above the navel. Main comorbidities and medications for diabetes and hypertension were also assembled. Information about pre-existing diagnosis of sleep apnea syndrome and treatment by continuous positive airway pressure (CPAP) was available.

Specifically, for the DietSleep study, the following data were collected: (1) the Berlin score, obtained from a 9-item questionnaire aiming to assess the risk of OSA [27], which is defined by a score of 2 points or more. Each sub-score of the Berlin questionnaire was analyzed separately. The sensitivity (Se) and specificity (Sp) of the Berlin questionnaire were assessed to screen severe OSA in the general population and were: Se = 76.9 and Sp = 72.7 [23]. (2) Daytime sleepiness was measured using the ESS. The ESS was considered as both a continuous and a categorical variable, with a threshold at 11; patients with ESS ≥11 were considered to have excessive daytime sleepiness. (3) Blood pressure was measured three times at each visit using an OMRON M6 Comfort blood pressure monitor under the supervision of trained dietician. These measures were performed twice during the study: once at the inclusion time and once at the final time of the RNPC program.

### 2.5. Statistical Analyses

Data were expressed as median and interquartile ranges for continuous values and as number and percent for qualitative values. Comparison between values at the start and the end of the treatment was performed using the Wilcoxon signed rank test for quantitative values and the McNemar test for qualitative values. Multivariable linear models were performed to assess the impact of the OSA-risk group on the evolution of anthropometric measures. Considered variables were age, gender, OSA-risk group, and the initial measure. Finally, a logistic regression model was performed to assess the probability of having high OSA risk after the weight-loss program, after adjusting for the main confounders (age, gender and waist circumference).

## 3. Results

### 3.1. Population

**Figure 2:**
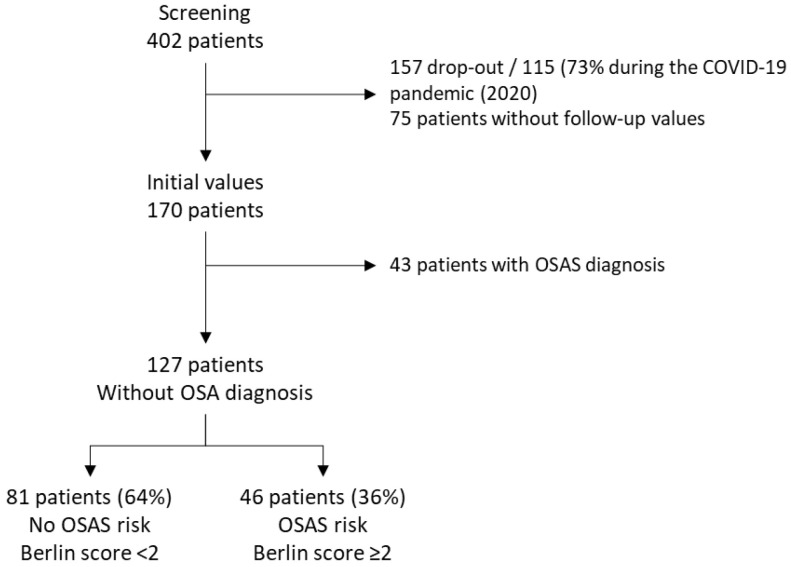
Flow chart of the study.

From 402 patients selected for the study, 127 patients were analyzed; 81 patients (64%) had no OSA risk (Berlin score < 2) and 46 patients (36%) had a risk of OSA (Berlin score ≥ 2) at baseline (Figure 2). There were no significant differences in age and sex between the included patients and those lost to follow-up.

At baseline, patients were mainly female (N = 109, 85.8%), with a median age of 52 years (interquartile range: 44–61), and they were more prone to hypertension and diabetes (Table 1). Anthropometric measures were significantly different between groups, with higher initial values for weight, BMI, waist circumference, percentages of fat mass, body water, and diastolic blood pressure in patients at risk of OSA compared to patients without an identified risk of OSA (Table 1).

### 3.2. Prevalence of Patients at Risk of OSA and Sleepy at Baseline

At the initiation of the program, the prevalence of patients with a high OSA risk was 36%. Regarding the different sub-scores of the Berlin questionnaire, patients with a Berlin score ≥2 had a higher prevalence of snoring (73.9%), sleepiness (43.5%), and comorbidities (91.3%) compared to patients without an identified baseline OSA risk.

Regarding the ESS, the score was higher for patients with OSA risk compared to patients without OSA risk: 7 (5; 10) vs. 6 (3; 8) (*p* < 0.01). The prevalence of patients with excessive daytime sleepiness (ESS score ≥11) tended to be higher in the group of patients with baseline OSA risk (19.6%) compared to patients without baseline OSA risk (9.9%) (*p* = 0.12) (Table 2).

### 3.3. Changes in Anthropometric Measures, OSA Risk, and Excessive Daytime Sleepiness between Initiation and End of the Program according to Initial OSA Risk

**Figure 3:**
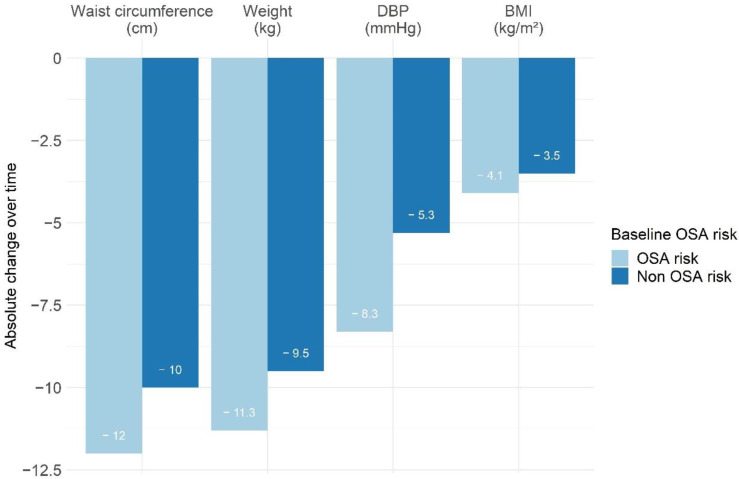
Non-adjusted significant absolute changes during the program in anthropometric markers of fat distribution associated with high cardiometabolic risk and blood pressure. DPB: diastolic blood pressure; BMI: body mass index. OSA: obstructive sleep apnea.

The median duration of the program was 5.6 months (3.8; 8.4). All anthropometric markers of fat distribution associated with high cardiometabolic risk decreased significantly during the program. Muscle mass percentage significantly increased in both groups. By comparing patients with or without baseline OSA risk, there was a significantly greater decrease in BMI and diastolic blood pressure, as well as a higher decrease in waist circumference (*p* = 0.06) in the group with baseline OSA risk compared to the group without baseline OSA risk (Table 3, Figure 3). The median percent of weight loss was 12.2% (8.9; 16.1) for all patients. Although there was a significant difference in absolute weight loss, the percentage of weight loss was not significantly different for both groups (*p* = 0.37). However, after multivariable adjustments to baseline values, these differences disappeared.

**Figure 4:**
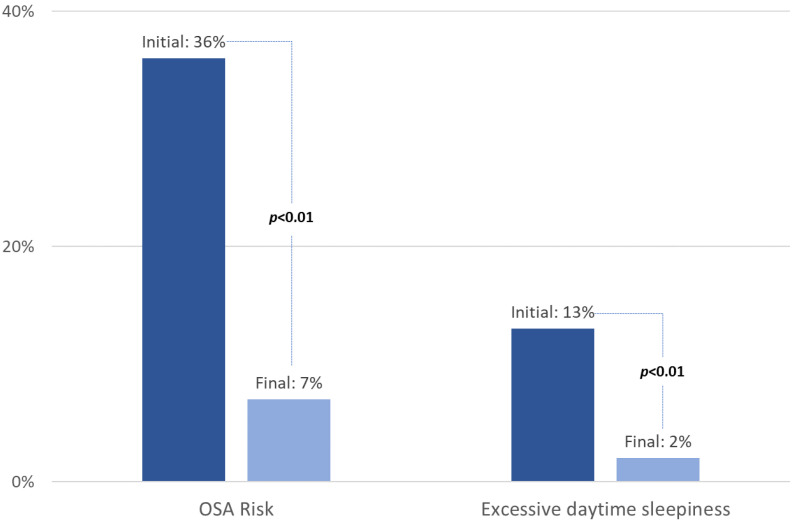
Changes in Berlin and ESS score proportions over time. OSA: obstructive sleep apnea. OSA risk: BERLIN score ≥ 2—Excessive daytime sleepiness: Epworth sleepiness scale ≥ 11.

There was a higher decrease in ESS score in the group of patients with baseline OSA risk (median decrease: −2 (−6; −1)) compared to patients without baseline OSA risk (median decrease: −1 (−3; 0)), *p* = 0.02. Patients with baseline OSA risk showed a more significant decrease regarding the Berlin score. The proportion of patients with a positive Berlin score decreased from 36% at baseline to 7% at the end of the program (*p* < 0.01), and the proportion of patients with excessive daytime sleepiness decreased from 13% at baseline to 2% at the end of the program (*p* < 0.01) (Figure 4).

### 3.4. Factor Associated with a Decrease in Berlin Score for Initial High Risk of OSA Patients

Regarding 47 patients who had a high risk of OSA at baseline, we utilized a logistic regression model to assess the determinants of a decrease in Berlin score. The reduction in waist circumference induced by waist low was significantly associated with a decrease in the Berlin score: for a one-centimeter decrease in waist circumference, the probability of normalizing the Berlin score at the end of the program increased by 19.8% (OR: 1.198 (1.021; 1.405), as adjusted for age and duration of the program.

## 4. Discussion

Our results showed a 36% prevalence rate of patients at high OSA risk at the initiation of the weight-loss program. Patients who had a high OSA risk exhibited more comorbidities and higher baseline values for anthropometric markers of fat distribution associated with a higher cardiometabolic risk and diastolic blood pressure. The subgroup initially at risk for OSA demonstrated a better response to the weight-loss program in terms of absolute values of improvement in adiposity indices compared to patients with non-OSA risk at baseline. However, the percentage of weight loss was similar in both groups; as reported by previous studies, the observed percentage of weight loss corresponded to a median decrease of 12.1% over time. By comparing these results to similar studies of OSA patients, such a weight loss is associated with a 50% decrease in OSA severity [5,28], which confirms the impact of a weight-loss program on the reduction in OSA risk in non-diagnosed OSA patients.

One originality of our cohort is the fact that we focused on overweight and obese patients rather than morbidly obese individuals, who are typically eligible for bariatric surgery [29]. Our results were consistent and comparable with previous studies [29,30]. After adjusting for baseline values, we found a similar evolution in anthropometric markers of fat distribution associated with high cardiometabolic risk in the specific subgroups with OSA risk versus non-OSA risk at baseline. Conversely, some previous studies have suggested that untreated OSA is associated with a lower decrease in ectopic fat or weight compared to patients without OSA [29,31,32]. A more systematic diagnosis of OSA and a combination of treatments are probably the most effective approaches. If an OSA recovery is demonstrated after weight-loss stabilization, then the primary therapy for OSA could be stopped.

Finally, in our study, patients included in the weight-loss program experienced a significant decrease in diastolic blood pressure. High diastolic blood pressure is typical in OSA, as it reflects a high level of sympathetic activity [33]. Our study demonstrated a considerable weight-loss effect on this marker of cardiovascular risk in the overall population, including the OSA-risk group.

The underdiagnosis of OSA in overweight and obese patients demonstrates that we need to develop alternative diagnosis pathways. Our results show that, for patients at OSA risk and without an OSA diagnosis, the Berlin score is a simple questionnaire with a high sensitivity but a lack of specificity, and it can be easily used by dieticians at the beginning of a weight-loss program. Moreover, a new home sleep test has also been developed, and it can be repeated over the course of weight loss; this tool should be disseminated in physicians’ offices and obesity clinics in the future for the better management of OSA [34,35,36].

Currently, only weight loss has been proven to solve OSA in the long term [37], while other overweight/obesity-associated metabolic and cardiovascular comorbidities generally coexist with OSA. Chirinos et al. compared the effect of combined CPAP and weight loss to CPAP or weight loss alone, and they showed that only combined interventions and weight loss induced significant decreases in the level of the inflammatory marker CRP, insulin resistance, and triglycerides [17]. In their systematic analysis of randomized studies comparing therapeutic versus sham CPAP intervention, Jullian-Desayes et al. failed to demonstrate that CPAP improves metabolic markers, most notably glucose, lipid, and insulin resistance levels, inflammatory markers, or metabolic syndrome in OSA patients [38]. Therefore, it is unrealistic to expect metabolic and/or cardiovascular ameliorations with CPAP, and we cannot consider it a therapy for OSA resolution, but only a means of avoiding its complications. There is an urgent need to systematically apply weight-loss strategies to patients at risk of OSA in order to prevent complications and improve metabolic and cardiovascular health. In our study, we consider only the effect of diet on weight loss without considering changes in physical activity. Physical exercise is important to take into account in a weight-loss program, as it has been shown to increase weight loss [18,39].

This study had several limitations. First, due to the COVID-19 pandemic, the sample size was more limited than expected, thus reducing the possibility of subgroups analyses. Regarding this point, we cannot rule out a possible selection bias due to the withdrawals. Second, the study used a questionnaire as a screening tool for OSA risk instead of polysomnography or polygraphy. Due to the limitations of the Berlin score—mainly patient misclassification—we cannot exclude that some patients suffering from mild-to-moderate OSA were classified in the non-OSA group, thus leading to an underestimation of OSA prevalence.

## 5. Conclusions

This study shows that there was a high prevalence of patients with OSA risk at the beginning of the RNPC weight-loss program. The results confirm that the program is associated with a significant decrease in OSA risk and excessive daytime sleepiness.

## Figures and Tables

**Table 1:** Description of baseline patient characteristics and anthropometric measures.

	All Population N = 127	Patients without Baseline OSA Risk N = 81 (55%)	Patients with Baseline OSA Risk N = 46 (45%)	*p*-Value
Age (years)	52 (44; 61)	53 (42; 62)	52 (45; 59)	0.29
Sex (female)	109 (85.8)	73 (90.1)	36 (78.3)	0.07
Tobacco consumption/yes	12 (9.4)	7 (8.6)	5 (10.9)	0.68
Arthrosis	29 (22.8)	15 (18.5)	14 (30.4)	0.12
Hypertension *	24 (18.9)	11 (13.6)	13 (28.3)	0.04
Diabetes *	5 (3.9)	1 (1.2)	4 (8.7)	0.04
Depression *	8 (6.3)	4 (4.9)	4 (8.7)	0.40
Anthropometric measures				
Weight (kg)	81.2 (74.2; 91.3)	78.8 (73.3; 84.4)	90.5 (79.7; 107)	<0.01
Height (cm)	165 (160; 170)	164 (161; 170)	165 (160; 171)	0.92
Body mass index (kg/m^2^)	29.7 (27.6; 34)	28.4 (27.3; 31.2)	33.2 (29.6; 36)	<0.01
Waist circumference (cm)	94 (89; 105)	92 (88; 100)	100 (91; 116)	<0.01
Fat mass (% of body weight)	41 (37.5; 43.4)	40.7 (37.1; 42.9)	42.4 (39.4; 45.2)	0.01
Muscle mass (% of body weight)	28.5 (26.4; 31.1)	28.9 (26.5; 31.1)	28.5 (27.2; 32)	0.50
Body water (% of body weight)	42.7 (40.4; 45)	43.3 (41.7; 45.9)	42 (40; 44.1)	0.01
Systolic blood pressure (mmHg) *	129.2 (117.8; 141.5)	126.7 (117.3; 136.3)	131 (121; 142.3)	0.12
Diastolic blood pressure (mmHg) *	85.3 (78; 93)	83.7 (77.3; 89.7)	86.2 (81.3; 93)	0.05

Data are in median (interquartile range) or number and percentage. * Missing values for hypertension, diabetes, and depression: N = 74; for systolic and diastolic blood pressure N = 2.

**Table 2:** Initial measures of Berlin and Epworth Sleepiness Scale for all population.

	All Population with Baseline Values N = 127	Patients without Baseline OSA Risk N = 81 (55%)	Patients with Baseline OSA Risk N = 46 (45%)	*p*-Value
Berlin score				
Snoring N,(%)	51 (40.2)	17 (21)	34 (73.9)	
Sleepiness N,(%)	27 (21.3)	7 (8.6)	20 (43.5)	
Comorbidities N,(%)	66 (52)	24 (29.6)	42 (91.3)	
Total Berlin score	1 (1; 2)	1 (0; 1)	2 (2; 2)	
Epworth Sleepiness Scale	6 (4; 9)	6 (3; 8)	7 (5; 10)	<0.01
Excessive daytime sleepiness (ESS ≥ 11) N,(%)	17 (13.4)	8 (9.9)	9 (19.6)	0.12

Data are in median (interquartile range) or number and percentage; ESS: Epworth Sleepiness Scale; OSA: Obstructive sleep apnea.

**Table 3:** Evolution of anthropometric measures, OSA risk, and ESS score over time during weight-loss program.

	Patients without Baseline OSA Risk N = 81 (64%)			Patients with Baseline OSA Risk N = 46 (36%)			*p*-Value ^$^	Adjusted *p*-Value
	Initial Measure	Final Measure	Delta (Final-Initial)	Initial Measure	Final Measure	Delta (Final-Initial)		
Duration of follow-up (months)			5.1 (3.7; 8)			5.7 (3.9; 9.2)	0.50	
Delta anthropometric measures								
Weight (kg)	79 (73.4; 84.7)	69.6 (64.1; 75.6)	−9.5 (−13.2; −6.8) *	89.6 (77.5; 107)	74.6 (65.5; 94.4)	−11.3 (−15.3; −8.5) *	0.02	0.69
Weight (% change)			−12.3 (−15.5; −8.8)			−11.9 (−17.1; 10.0)	0.37	
Body mass index (kg/m^2^)	28.6 (27.3; 31.3)	25.3 (23.6; 27.6)	−3.5 (−4.8; −2.6) *	33.2 (29.5; 36)	28.8 (25.4; 31.1)	−4.1 (−5.7; −3.3) *	0.02	0.98
Waist circumference (cm)	92.5 (88; 100)	81 (77; 86)	−10 (−16; −8) *	100 (90; 116)	87 (79; 99)	−12 (−19; −10) *	0.06	0.23
Fat mass (%)	40.7 (37; 43)	36.4 (32.4; 39.2)	−4.3 (−5.6; −3) *	42.3 (39.4; 45.2)	37.4 (35.1; 42)	−3.7 (−5.4; −2.4) *	0.42	0.42
Muscle mass (%)	28.9 (26.5; 31.2)	30.7 (27.7; 32.2)	1.3 (0.9; 1.9) *	28.5 (27.2; 32)	29.8 (28.7; 32.6)	1.5 (0.8; 2.2) *	0.69	<0.01 *
Body water (%)	43.4 (41.7; 46)	46.4 (44.5; 49.6)	3.1 (2.2; 4.1) *	42.1 (40; 44.1)	45.8 (42.3; 47.4)	2.8 (2; 3.9) *	0.61	0.64
Systolic blood pressure	125.8 (117; 136)	118.3 (108; 125)	−10.3 (−17.3; −3) *	131 (121; 145)	119.7 (110; 128)	−13.3 (−22.7; −4.7) *	0.22	0.46
Diastolic blood pressure	83.5 (77; 89)	78.3 (71; 84)	−5.3 (−10.3; −0.3) *	86.3 (81; 93)	78 (73; 84)	−8.3 (−13; −1.7) *	0.05	0.18
Berlin score	1 (0; 1)	0 (0; 0)	0 (−1; 0) *	2 (2; 2)	1 (0; 1)	−1 (−2; −1) *	<0.01	
Epworth Sleepiness Scale	6 (3; 8)	4 (2; 5.5)	−1 (−3; 0) *	7 (5; 10)	4 (2; 6)	−2 (−6; −1) *	0.02	

Data are in median (interquartile range) or number and percentage. ^$^: overall *p* value from Mann–Whitney test which compare delta values between groups (with or without baseline OSA risk).—adjusted *p* values are *p* values for Berlin score status at baseline adjusted on age, sex, and baseline values. * Significant paired comparison of values before and after RNPC program^®^.

## Data Availability

The data presented in this study are available on request from the corresponding author. The data are not publicly available due to GRPD.
